# Detection of diverse carbapenem and multidrug resistance genes and high-risk strain types among carbapenem non-susceptible clinical isolates of target gram-negative bacteria in Kenya

**DOI:** 10.1371/journal.pone.0246937

**Published:** 2021-02-22

**Authors:** Lillian Musila, Cecilia Kyany’a, Rosslyn Maybank, Jason Stam, Valerie Oundo, Willie Sang

**Affiliations:** 1 Department of Emerging Infectious Diseases, United States Army Medical Research Directorate-Africa, Nairobi, Kenya; 2 Multidrug-Resistant Organism Repository and Surveillance Network, Bacterial Diseases Branch, Walter Reed Army Institute of Research, Silver Spring, Maryland, United States of America; 3 Center for Microbiology, Kenya Medical Research Institute, Nairobi, Kenya; Nitte University, INDIA

## Abstract

Carbapenem-resistant gram-negative bacteria are an increasingly significant clinical threat globally. This risk may be underestimated in Kenya as only four carbapenemase genes in three bacterial species have been described. The study aimed to understand the antibiotic resistance profiles, genes, sequence types, and distribution of carbapenem-resistant gram-negative bacteria from patients in six hospitals across five Kenyan counties by bacterial culture, antibiotic susceptibility testing, and whole-genome sequence analysis. Forty-eight, non-duplicate, carbapenem non-susceptible, clinical isolates were identified across the five counties (predominantly in Nairobi and Kisii): twenty-seven *Acinetobacter baumannii*, fourteen *Pseudomonas aeruginosa*, three *Escherichia coli*, two *Enterobacter cloacae*, and two *Klebsiella pneumoniae*. All isolates were non-susceptible to β-lactam drugs with variable susceptibility to tigecycline (66%), minocycline (52.9%), tetracycline (29.4%), and levofloxacin (22.9%). Thirteen *P*. *aeruginosa* isolates were resistant to all antibiotics tested. Eleven carbapenemase genes were identified: *bla*_NDM-1,_ *bla*_OXA-23, -58, -66, -69, and -91_ in *A*. *baumannii (*STs 1, 2, 164 and a novel ST1475), *bla*_NDM-1_ in *E*. *cloacae* (STs 25,182), *bla*_NDM-1_, *bla*_VIM-1and -6_, *bla*_OXA-50_ in *P*. *aeruginosa* (STs 316, 357, 654, and1203), *bla*_OXA-181,_ *bla*_NDM-1_ in *K*. *pneumoniae* (STs 147 and 219), and *bla*_NDM-5_ in *E*. *coli* (ST164). Five *A*. *baumannii* isolates had two carbapenemases, *bla*_NDM-1,_ and either *bla*_OXA-23_ (4) or *bla*_OXA-58_ (1). AmpC genes were detected in *A*. *baumannii (bla*_ADC-25_), *E*. *cloacae* (*bla*_DHA-1 and_ *bla*_ACT-6, 16_), and *K*. *pneumoniae* (bla_CMY_). Significant multiple-drug resistant genes were the pan-aminoglycoside resistance16srRNA methyltransferase *arm*A, *rmt*B, *rmt*C, and *rmt*F genes. This study is the first to report *bla*_OXA-420, -58, -181, VIM-6,_ and *bla*_NDM-5_ in Kenyan isolates. High-risk STs of *A*. *baumannii* (ST1475, ST2), *E*. *cloacae* ST182, *K*. *pneumoniae* ST147, *P*. *aeruginosa* (ST357, 654), and *E*. *coli* ST167, ST648 were identified which present considerable therapeutic danger. The study recommends urgent carbapenem use regulation and containment of high-risk carbapenem-resistant bacteria.

## Introduction

Multidrug resistance among clinically significant gram-negative bacteria (GNB) (*Escherichia coli*, *Klebsiella pneumoniae*, Enterobacter *spp*. *Pseudomonas aeruginosa*, and *Acinetobacter baumannii*) has led to increased morbidity and an estimated 40% mortality in developing countries [1, 2]. These adverse outcomes are due to treatment options being limited to expensive, often unavailable, last-line drugs such as tigecycline. Carbapenems are an important β-lactam drug class used to treat serious multidrug-resistant bacterial infections. Therefore, the global increase in carbapenem resistance (CR) has been recognized as a severe health threat [3].

CR is mediated primarily by the expression of chromosomally-encoded or plasmid-encoded carbapenemases that fall into three classes: Class A (e.g., *K*. *pneumoniae* carbapenemases (KPC), Class B (e.g., New Delhi Metallo-β-lactamase (NDM), the Verona integrin-encoded Metallo-β-lactamase (VIM), and Imipenemase (IMP), and Class D (e.g.OXA-48 and -181) [4]. Secondary mechanisms for CR are the constitutive over-production of AmpC and changes in permeability due to loss or down-regulation of porins [5].

The global spread of CR is due to the carriage of CR genes on mobile genetic elements (plasmids, transposons, and integrons). These elements also harbor genes that confer resistance against multiple antibiotic classes [6, 7]. With the increased use of carbapenems, the prediction is that GNB will evolve to accumulate multiple CR genes and mechanisms of resistance [8]. This further increases the likelihood and clinical threat of the emergence of extensively drug-resistant (XDR) GNB. Growing global diversity and distribution of CR genes, which have been described extensively [9–12], and the identification of high-risk XDR strain types (STs) associated with outbreaks, further anchors this prediction. These high-risk clones include the international clone I-III [13] of *A*. *baumannii*, ST235, ST357, and ST664 of *P*. *aeruginosa*, ST258, and ST307 of *K*. *pneumoniae* [14, 15] and ST78 of *Enterobacter cloacae* [16].

Carbapenem resistance is a growing problem in Africa, with a reported prevalence ranging from <1% to 60% among GNB [17–19]. Recent studies in Tanzania and Uganda have shown that this prevalence could be as high as 22.4–35% with the circulation of CR genes *bla*_VIM_, *bla*_OXA-48_, *bla*_IMP_, *bla*_KPC,_ and *bla*_NDM-1_ predominantly among *K*. *pneumonia* and *P*. *aeruginosa* [20, 21]. In Kenya, only the carbapenemase genes *bla*_NDM-1_ in *K*. *pneumoniae*, *P*. *aeruginosa and A*. *baumannii*, *bla*_OXA-23_ in *A*. *baumannii* [22–24], *bla*_SPM_ in *K*. *pneumoniae* [25], and *bla*_VIM-2_ in *P*. *aeruginosa* [26] have been identified. These are from hospitals in three Kenyan counties: Nairobi, Kiambu, and Kilifi. With increasing carbapenem use in Kenya because of the high levels of ESBL-producing Enterobacteriaceae [27–29], a concomitant rise in CR is expected that warrants close monitoring.

This study aimed to address the limited data available on the diversity and distribution of carbapenemase and other antibiotic resistance genes and strain types of clinically relevant C-NS GNB. The focus was on five bacterial species: *E*. *coli*, *K*. *pneumoniae*, *A*. *baumannii*, *P*. *aeruginosa*, and Enterobacter *spp*. from clinical isolates in hospitals from five Kenyan counties. The research demonstrated a greater variety and distribution of carbapenemase genes in Kenya than previously recognized. It further identified the therapeutic risks associated with CR infections and identified high-risk, multidrug-resistant strains. These findings underscore the importance of targeted surveillance and control of C-NS GNB.

## Materials and methods

### Ethics and approvals

This study was approved by the Kenya Medical Research Institute (KEMRI) Scientific and Ethics Review Unit (#2767), the Walter Reed Army Institute of Research (WRAIR) Institutional Review Board (#2089), and the U.S. Army Medical Research and Materiel Command, Office of Research Protection, Human Research Protections Office (USAMRMC ORP HRPO) (Log#A-18129). The investigators adhered to the policies for the protection of human subjects as prescribed in AR 70–25. Study participants provided written consent to participate in the study.

### Isolate culture and antimicrobial susceptibility testing

*A*. *baumannii*, *P*. *aeruginosa*, *Enterobacter spp*., *K*. *pneumoniae*, and *E*. *coli* isolates were obtained from patients with skin and soft tissue infections (SSTI) and urinary tract infections (UTI). These patients were enrolled in an antimicrobial resistance surveillance study conducted between 2015 to 2018 in six hospitals in 5 Kenyan counties: Nairobi, Kisumu, Kisii, Kilifi, and Kericho. The Nairobi and Kisii county hospitals are full-service teaching and referral hospitals with large inpatient capacities. In contrast, the other four hospitals are county or sub-county level hospitals that offer primary outpatient care, surgical, laboratory, maternity, and inpatient services.

Demographic and clinical information was collected from each study subject. The isolates were identified, and antimicrobial susceptibility testing performed on the VITEK 2^®^ automated platform (bioMérieux, Marcy l’Etoile, France) using the GN-ID and XN05 AST cards. Only one isolate was tested on a Microscan (Beckman Coulter, Indianapolis, USA). The antibiotics reported in this study are ceftriaxone, cefepime, ticarcillin-clavulanic acid, piperacillin, meropenem, levofloxacin, tetracycline, tigecycline, and minocycline. Minimum inhibitory concentration (MIC) data for each organism were interpreted according to the Clinical and Laboratory Standards Institute guidelines (2015) and the VITEK 2^®^ Advanced Expert System (AES). All non-duplicate carbapenem non-susceptible (C-NS) isolates, both intermediate and resistant to meropenem, detected from 2015 to 2018 were selected for further analysis.

### Genomic characterization and strain typing

Whole-genome sequencing was performed on C-NS isolates to detect the antibiotic resistance genes. DNA was extracted using the DNeasy UltraClean Microbial Kit (Qiagen, Germantown, MD, USA), and libraries constructed using the KAPA HyperPlus Library preparation kit (Roche Diagnostics, Indianapolis, Indiana, USA). These libraries were quantified using the KAPA Library Quantification Kit–Illumina/Bio-Rad iCycler™ (Roche Diagnostics, Indianapolis, Indiana, USA) and sequenced with a MiSeq Reagent Kit v3 (600 cycles) on an Illumina MiSeq desktop sequencer (Illumina Inc., San Diego, CA, USA). Species identification and contamination detection were performed from sequencing reads using Kraken2 [30]. The reads were trimmed for adapter content and quality, followed by *de novo* assembly using Newbler v2.7. Antimicrobial resistance genes were annotated using ResFinder v3.2 [31], and MLST assignment performed using parsed nucleotide *BLA*ST results against the relevant schema hosted by PubMLST [32]. Whole-genome sequences are deposited in GenBank^®^ BioProject IDs PRJNA636771 and PRJNA555206. For isolates with no assigned sequence types (STs), genome assemblies were submitted to PubMLST for strain assignment using the BIGSdb software [33].

### Data management and analysis

Patient data (clinical and demographic) and the antimicrobial susceptibility (AS) data for each isolate extracted from the automated platforms were compiled in a Microsoft Access database. These compiled data were transferred to Microsoft Excel for descriptive statistical analysis with frequency distributions and the results summarized in tables.

## Results

### Demographic and clinical characteristics of subjects

C-NS isolates were identified from forty-eight subjects in all the five study counties. Demographic and clinical information was available for all but one subject, KPA1. The majority of the C-NS isolates were from subjects in the larger referral hospitals in Nairobi (29, 60.4%) and Kisii counties (11, 22.9%). Approximately 69% of the isolates were from male subjects, 87% were inpatient, and 64% were healthcare-associated infections (HAI) per the CDC/NHSN Surveillance Definition of Healthcare-Associated Infection [34]. Approximately 64% (30/47) of isolates were from SSTI, including ear infections, injury wounds, cellulitis, abscesses, burns, bedsores, cancer lesions, amputation sites, and surgical site infections. The remaining 36% (17/47) of the isolates were from inpatient subjects with UTIs, of which 70.5% (12/17) were catheter-associated (Table 1).

**Table 1:** Characteristics of subjects from whom C-NS isolates were obtained.

Isolate ID	County	Sex	Patient type	Infection type	Infection site	Infection acquisition
***Acinetobacter baumannii***						
**KAB1**	Nairobi	F	Inpatient	SSTI	Leg	CAI
**KAB2**	Nairobi	M	Inpatient	UTI	Catheter	CAI
**KAB3**	Nairobi	M	Inpatient	UTI	Catheter	HAI
**KAB4**	Nairobi	M	Inpatient	UTI	Catheter	HAI
**KAB5**	Nairobi	M	Inpatient	SSTI	Catheter	HAI
**KAB6**	Nairobi	M	Inpatient	SSTI	Leg	unclear
**KAB7**	Nairobi	M	Inpatient	SSTI	Leg	HAI
**KAB8**	Kisumu	F	Outpatient	UTI	Catheter	HAI
**KAB9**	Nairobi	M	Inpatient	UTI	na	HAI
**KAB10**	Nairobi	F	Inpatient	SSTI	Leg	HAI
**KAB11**	Nairobi	F	Outpatient	SSTI	Foot	HAI
**KAB12**	Kericho	F	Inpatient	SSTI	Leg	CAI
**KAB13**	Kisii	F	Inpatient	SSTI	Leg	HAI
**KAB14**	Kisii	M	Inpatient	SSTI	Leg	CAI
**KAB15**	Kisumu	F	Inpatient	UTI	Catheter	HAI
**KAB16**	Kisumu	F	Inpatient	SSTI	Trunk	HAI
**KAB17**	Kisii	F	Inpatient	SSTI	Breast	CAI
**KAB18**	Kisii	M	Inpatient	SSTI	Thigh	HAI
**KAB19**	Kisii	M	Inpatient	SSTI	Scrotum	CAI
**KAB20**	Kisii	F	Inpatient	SSTI	Buttocks	CAI
**KAB21**	Kisii	F	Inpatient	SSTI	Breast	CAI
**KAB22**	Kisii	M	Outpatient	SSTI	Leg	CAI
**KAB23**	Kisii	M	Inpatient	SSTI	Foot	CAI
**KAB24**	Kisii	F	Inpatient	SSTI	Abdomen	HAI
**KAB25**	Kericho	M	Inpatient	SSTI	Thigh	CAI
**KAB26**	Kericho	M	Inpatient	SSTI	Thigh	CAI
**KAB27**	Kisumu	M	Inpatient	SSTI	Penis	CAI
***Enterobacter cloacae***						
**KEB1**	Nairobi	M	Inpatient	UTI	Catheter	HAI
**KEB2**	Nairobi	M	Inpatient	SSTI	Arm	HAI
***Escherichia coli***						
**KEC1**	Kisii	F	Inpatient	SSTI	Back	CAI
**KEC2**	Nairobi	M	Inpatient	UTI	na	HAI
**KEC3**	Nairobi	F	Inpatient	SSTI	na	HAI
***Klebsiella pneumoniae***						
**KKPI**	Nairobi	M	Outpatient	SSTI	Leg	CAI
**KKP2**	Kilifi	M	Outpatient	SSTI	Leg	CAI
***Pseudomonas aeruginosa***						
**KPA1**	Nairobi	na	na	na	na	na
**KPA2**	Nairobi	M	Inpatient	UTI	Catheter	HAI
**KPA3**	Nairobi	M	Inpatient	UTI	Clean-catch	CAI
**KPA4**	Nairobi	M	Inpatient	UTI	Catheter	HAI
**KPA5**	Nairobi	M	Inpatient	UTI	na	HAI
**KPA6**	Nairobi	M	Inpatient	UTI	Catheter	HAI
**KPA7**	Nairobi	M	Inpatient	SSTI	Leg wound	HAI
**KPA8**	Nairobi	M	Inpatient	SSTI	na	HAI
**KPA9**	Nairobi	M	Inpatient	SSTI	na	HAI
**KPA10**	Nairobi	M	Inpatient	UTI	Catheter	HAI
**KPA11**	Nairobi	M	Inpatient	SSTI	Buttocks	HAI
**KPA12**	Nairobi	M	Inpatient	UTI	na	HAI
**KPA13**	Nairobi	M	Inpatient	UTI	na	HAI
**KPA14**	Nairobi	M	Outpatient	UTI	Catheter	CAI

SSTI, skin and soft tissue; UTI, urinary tract infections; CAI, community-acquired infection; HAI, healthcare-associated infection; M, male; F, female; na, information not available.

### Identity and antimicrobial susceptibility patterns of carbapenem-non-susceptible isolates

The forty-eight isolates selected based on their non-susceptibility to meropenem (Table 2) were: twenty-seven *A*. *baumannii*, fourteen *P*. *aeruginosa*, three *E*. *coli*, two *E*. *cloacae*, and two *K*. *pneumoniae* (Tables 1 and 2). All isolates were resistant to meropenem except for three *A*. *baumannii* isolates (KAB14, 15, 16). These had intermediate resistance (MIC = 8), or AES interpreted intermediate resistance (MIC = 4). All the isolates were resistant to penicillin, penicillin/β-lactamase inhibitor combination, and cephalosporin drugs tested. The only exception was a single isolate (KPA3) that was susceptible to cefepime. The isolates were most susceptible to tigecycline (66%), followed by minocycline (52.9%), tetracycline (29.4%), and levofloxacin (22.9%). All fourteen *P*. *aeruginosa* isolates, two *A*. *baumannii* (KAB4, KAB6) and one *K*. *pneumoniae* (KKP1) isolates were resistant to all antibiotics tested. Eight *A*. *baumannii* isolates (KAB1-3, 7, 9–11, 13, 25–27), three *E*. *coli (*KEC1, 2, 3), and one *E*. *cloacae* (KEB1) were resistant to all but tigecycline.

**Table 2:** Antimicrobial susceptibility profiles of the C-NS bacterial isolates.

Isolate ID	Antibiotic susceptibility test MICs in μg/mL (Interpretation)								
	MEM	CRO	PIP	TIM	FEP	LVX	TET	MIN	TGC
***Acinetobacter baumannii (n = 27)***									
KAB1	> = 16 (R)	> = 64 (R)	> = 128 (R)	> = 128 (R)	> = 64 (R)	> = 8 (R)	> = 16 (R)	4 (S)	1 (S)
KAB2	> = 16 (R)	> = 64 (R)	> = 128 (R)	> = 128 (R)	> = 64 (R)	> = 8 (R)	> = 16 (R)	4 (S)	1 (S)
KAB3	> = 16 (R)	> = 64 (R)	> = 128 (R)	nd	> = 64 (R)	> = 8 (R)	> = 16 (R)	4 (S)	1 (S)
KAB4	> = 16 (R)	> = 64 (R)	> = 128 (R)	> = 128 (R)	> = 64 (R)	> = 8 (R)	> = 16 (R)	> = 16 (R)	> = 8 (R)
KAB5	> = 16 (R)	> = 64 (R)	> = 128 (R)	> = 128 (R)	> = 64 (R)	> = 8 (R)	> = 16 (R)	4 (S)	2 (S)
KAB6	> = 16 (R)	> = 64 (R)	> = 128 (R)	> = 128 (R)	> = 64 (R)	> = 8 (R)	> = 16 (R)	> = 16 (R)	4 (I)
KAB7	> = 16 (R)	> = 64 (R)	> = 128 (R)	> = 128 (R)	> = 64 (R)	> = 8 (R)	> = 16 (R)	8 (I)	2 (S)
KAB8	> = 16 (R)	> = 64 (R)	> = 128 (R)	> = 128 (R)	> = 64 (R)	0.5 (S)	> = 16 (R)	< = 1 (S)	2 (S)
KAB9	> = 16 (R)	> = 64 (R)	> = 128 (R)	> = 128 (R)	> = 64 (R)	> = 8 (R)	> = 16 (R)	8 (I)	2 (S)
KAB10	> = 16 (R)	> = 64 (R)	> = 128 (R)	> = 128 (R)	> = 64 (R)	> = 8 (R)	> = 16 (R)	8 (I)	2 (S)
KAB11	> = 16 (R)	> = 64 (R)	> = 128 (R)	> = 128 (R)	> = 64 (R)	> = 8 (R)	> = 16 (R)	8 (I)	2 (S)
KAB12	> = 16 (R)	> = 64 (R)	> = 128 (R)	> = 128 (R)	> = 64 (R)	> = 8 (R)	> = 16 (R)	2 (S)	2 (S)
KAB13	> = 16 (R)	> = 64 (R)	> = 128 (R)	> = 128 (R)	> = 64 (R)	4 (R)	> = 16 (R)	> = 16 (R)	2 (S)
KAB14	8 (I)	> = 64 (R)	> = 128 (R)	> = 128 (R)	> = 64 (R)	4 (R)	> = 16 (R)	< = 1 (S)	< = 0.5 (S)
KAB15	4 (I*)	> = 64 (R)	> = 128 (R)	> = 128 (R)	> = 64 (R)	4 (R)	2 (S)	< = 1 (S)	< = 0.5 (S)
KAB16	8 (I)	> = 64 (R)	> = 128 (R)	> = 128 (R)	> = 64 (R)	4 (R)	4 (S)	< = 1 (S)	< = 0.5 (S)
KAB17	> = 16 (R)	> = 64 (R)	> = 128 (R)	> = 128 (R)	> = 64 (R)	< = 0.12 (S)	4 (S)	< = 1 (S)	1 (S)
KAB18	> = 16 (R)	> = 64 (R)	> = 128 (R)	> = 128 (R)	> = 64 (R)	< = 0.12 (S)	4 (S)	< = 1 (S)	1 (S)
KAB19	> = 16 (R)	> = 64 (R)	> = 128 (R)	> = 128 (R)	> = 64 (R)	0.25 (S)	8(I)	2 (S)	4 (I)
KAB20	> = 16 (R)	> = 64 (R)	> = 128 (R)	> = 128 (R)	> = 64 (R)	< = 0.12 (S)	4 (S)	< = 1 (S)	< = 0.5 (S)
KAB21	> = 16 (R)	> = 64 (R)	> = 128 (R)	> = 128 (R)	> = 64 (R)	< = 0.12 (S)	4 (S)	< = 1 (S)	< = 0.5 (S)
KAB22	> = 16 (R)	> = 64 (R)	> = 128 (R)	> = 128 (R)	> = 64 (R)	< = 0.12 (S)	4 (S)	2 (S)	1 (S)
KAB23	> = 16 (R)	> = 64 (R)	> = 128 (R)	> = 128 (R)	> = 64 (R)	< = 0.12 (S)	4 (S)	< = 1 (S)	1 (S)
KAB24	> = 16 (R)	> = 64 (R)	> = 128 (R)	> = 128 (R)	> = 64 (R)	< = 0.12 (S)	4 (S)	2 (S)	< = 0.5 (S)
KAB25	> = 16 (R)	> = 64 (R)	> = 128 (R)	> = 128 (R)	> = 64 (R)	4 (R)	> = 16 (R)	> = 16 (R)	1 (S)
KAB26	> = 16 (R)	> = 64 (R)	> = 128 (R)	> = 128 (R)	> = 64 (R)	4 (R)	> = 16 (R)	> = 16 (R)	2 (S)
KAB27	> = 16 (R)	> = 64 (R)	> = 128 (R)	> = 128 (R)	> = 64 (R)	4 (R)	8 (I)	> = 16 (R)	2 (S)
***Enterobacter cloacae (n = 2)***									
KEB1	> = 16 (R)	> = 64 (R)	> = 128 (R)	> = 128 (R)	> = 64 (R)	> = 8 (R)	> = 16 (R)	> = 16 (R)	2 (S)
KEB2	> = 16 (R)	> = 64 (R)	> = 128 (R)	> = 128 (R)	32 (R)	0.25 (S)	4 (S)	> = 16 (R)	2 (S)
***Escherichia coli (n = 3)***									
KEC1	8 (R)	> = 64 (R)	> = 128 (R)	> = 128 (R)	> = 64 (R)	> = 8 (R)	> = 16 (R)	> = 16 (R)	< = 0.5 (S)
KEC2	8 (R)	> = 64 (R)	> = 128 (R)	> = 128 (R)	> = 64 (R)	> = 8 (R)	> = 16 (R)	> = 16 (R)	< = 0.5 (S)
KEC3	8 (R)	> = 64 (R)	> = 128 (R)	> = 128 (R)	> = 64 (R)	> = 8 (R)	> = 16 (R)	> = 16 (R)	< = 0.5 (S)
***Klebsiella pneumoniae(n = 2)***									
KKPI	> = 16 (R)	> = 64 (R)	> = 128 (R)	> = 128 (R)	> = 64 (R)	> = 8 (R)	> = 16 (R)	> = 16 (R)	> = 8 (R)
KKP2	> = 16 (R)	> = 64 (R)	> = 128 (R)	> = 128 (R)	16 (R)	1 (S)	> = 16 (R)	4 (S)	< = 0.5 (S)
***Pseudomonas aeruginosa(n = 14)***									
KPA1	> = 8 (R)^**#**^	nd	>64 (R) ^**#**^	nd	>8 (R) ^**#**^	> = 4 (R) ^**#**^	nd	nd	nd
KPA2	> = 16 (R)	nd	> = 128 (R)	> = 128 (R)	> = 64 (R)	> = 8 (R)	nd	nd	> = 8 (R)
KPA3	> = 16 (R)	nd	64 (R)	> = 128 (R)	8 (S)	> = 8 (R)	nd	nd	> = 8 (R)
KPA4	> = 16 (R)	nd	> = 128 (R)	> = 128 (R)	> = 64 (R)	> = 8 (R)	nd	nd	> = 8 (R)
KPA5	> = 16 (R)	nd	> = 128 (R)	> = 128 (R)	> = 64 (R)	> = 8 (R)	nd	nd	> = 8 (R)
KPA6	> = 16 (R)	nd	> = 128 (R)	> = 128 (R)	> = 64 (R)	> = 8 (R)	nd	nd	> = 8 (R)
KPA7	> = 16 (R)	nd	> = 128 (R)	> = 128 (R)	> = 64 (R)	> = 8 (R)	nd	nd	> = 8 (R)
KPA8	> = 16 (R)	nd	> = 128 (R)	> = 128 (R)	> = 64 (R)	> = 8 (R)	nd	nd	> = 8 (R)
KPA9	> = 16 (R)	nd	> = 128 (R)	> = 128 (R)	> = 64 (R)	> = 8 (R)	nd	nd	> = 8 (R)
KPA10	> = 8 (R)	nd	> = 128 (R)	> = 128 (R)	> = 64 (R)	> = 8 (R)	nd	nd	> = 8 (R)
KPA11	> = 8 (R)	nd	> = 128 (R)	> = 128 (R)	> = 64 (R)	> = 8 (R)	nd	nd	> = 8 (R)
KPA12	> = 16 (R)	nd	> = 128 (R)	> = 128 (R)	> = 64 (R)	> = 8 (R)	nd	nd	> = 8 (R)
KPA13	> = 16 (R)	nd	> = 128 (R)	> = 128 (R)	> = 64 (R)	> = 8 (R)	nd	nd	> = 8 (R)
KPA14	> = 8 (R)	nd	> = 128 (R)	> = 128 (R)	> = 64 (R)	> = 8 (R)	nd	nd	> = 8 (R)
**No. tested**	48	34	48	46	48	48	34	34	47
**No. susceptible (S)**	0	0	0	0	1	11	10	18	31
**% S**	**0.0**	**0.0**	**0.0**	**0**	**2.1**	**22.9**	**29.4**	**52.9**	**66.0**

MIC values that indicate non-susceptibility to the antibiotic are shaded grey.

The %S is the number of susceptible isolates as a percentage of all isolates tested for each antibiotic.

MIC, minimum inhibitory concentration; CRO, ceftriaxone; FEP, cefepime; TIM, ticarcillin-clavulanic acid; PIP, piperacillin; MEM, meropenem; LVX, levofloxacin; TET, tetracycline; TGC, tigecycline; MIN, minocycline; nd, antibiotics not tested.

*Non-susceptibility inferred by the VITEK 2^®^ AES system.

^#^ AST performed on a Microscan platform.

### Carbapenemase genes detected across bacterial species

Eleven different carbapenemases from the class B Metallo-β-lactamases (*bla*_NDM-1_, *bla*_NDM-5_, *bla*_VIM-2,_ and *bla*_VIM-6)_ and the Class D oxacillinases (*bla*_OXA-23_, *bla*_OXA-50,_ *bla*_OXA-58_, *bla*_OXA-181,_ *bla*_OXA-66,_ *bla*_OXA-69,_ *bla*_OXA-91_) were detected in the 48 isolates (Tables 3 and 4).

**Table 3:** The diversity of carbapenemase genes identified among the bacterial species.

Carbapenemase Ambler Class	B: Metallo- β lactamases				Class D: Oxacillinases						
**Bacterial species**	***NDM-1***	***NDM-5***	***VIM-1***	***VIM-6***	***OXA-23***	***OXA-58***	***OXA-66***	***OXA-69***	***OXA-91***	***OXA-181***	***OXA-50***
*A*. *baumannii*	7				23	1	11	13	2		
*E*. *cloacae*	2										
*P*. *aeruginosa*	11		1	2							1
*K*. *pneumoniae*	1									1	
*E*. *coli*		3									
No of occurrences	21	3	1	2	23	1	11	13	2	1	1
(n = 79) **(%)**	(**26.6)**	(**3.8)**	(**1.3)**	(**2.5)**	(**29.1)**	(**1.3)**	(**13.9)**	(**16.5)**	(**2.5)**	(**1.3)**	(**1.3)**

NDM, New Delhi Metallo-β-lactamase; OXA, oxacillinase; VIM, Verona integron-encoded Metallo-β-lactamase.

**Table 4:** Distribution of drug resistance genes among the C-NS species and strain types.

Isolate IDs	Species (No. of isolates)	ST	Carbapenemases	Penicillinases and β-lactamases *	Aminoglycoside resistance genes ^#^	Efflux genes	Other significant antibiotic resistance genes
KAB18-20	AB (3)	1	OXA-23, 69	**ADC-25**			*sul*2
KAB17,21–24	AB (5)	1	OXA-23, 69	**ADC-25**	*ant(2’’)-Ia*		*sul2*
KAB25, 26,27	AB (3)	1	NDM-1, OXA-23, 69	ADC-191	*aac(3)-I*, *aad*A1, *adeC aph(3’)-Ia*, *aph(3’’)-Ib*, *aph(3’)-Via*, *aph(6)-Id ant(3’’)-Iia*	*amv*A	*ble*, *dfr*A1, *sat*2, *sul*1/2, *tet*B
KAB16	AB (1)	1	NDM-1, OXA-69	**ADC-25,** CARB-16	*aad*A1, *aph(3’)-Ia*, *aph(3’)-VI*		*mph*E, *msr*E, *sul*2, *dfr*A1
KAB1-5,7,9, 10	AB (8)	2	OXA-23, OXA-66	**ADC-25,** TEM-1D	***arm*A**, *aph(3’’)-Ib*, *aph(3’)-Ia*, *aph(6)-Id*		*mph*E, *msr*E, *tet*B, *sul*2
KAB6,11	AB (2)	2	OXA-23, OXA-66	**ADC-25,** TEM-1D	***arm*A** *aph(3’’)-Ib*, *aph(3’)-Ia*, *aph(6)-Id*		*mph*E, *msr*E, *tet*B
KAB12	AB (1)	2	OXA-23, OXA-66	**ADC-25,** TEM-1D	*aac(3)-Ia*, *aad*A1,*aph(3’)-Ia*		*mph*E, *msr*E, *sul*1/2
KAB13	AB (1)	1475	NDM-1, OXA-23, 69	**ADC-25**	*aac(3)-Ia*, *aad*A1, *aph(3’’)-Ib*, *aph(3’)-Ia*, *aph(3’)-Via*, *aph(6)-Id*		*dfr*A1, *sul*1/2, *tet*B
KAB8	AB (1)	374	NDM-1	ADC-26, OXA-259, CARB-16	*aad*A1, *ant(2’’)-Ia*, *ant(3’’)-Iia*, *aph(3’)-Ia*	*amv*A	*ble*, *dfr*A1, *mph*E, *msr*E, *sat2*, *sul*2, *tet*39
KAB14	AB (1)	164	NDM-1, OXA-58, 91	CARB-16	*aac(3)-Iid*, *ant(2’’)-Ia*, *aph(3’’)-Ib*, *aph(3’)-Ia*, *aph(3’)-Via*, *aph(6)-Id*		*flo*R, *mph*E, *msr*E, *tet*39, *sul2*
KAB15	AB (1)	164	OXA-420, 91	CARB-16	*ant(2’’)-Ia*, *aph(3’’)-Ib*, *aph(3’)-Ia*, *aph(6)-Id*		*dfr*A20, *sul2*
KEB1	ECL (1)	182	NDM-1	**ACT-16, DHA-1** TEM-1B, OXA-1	***rmt*C,** *aac(6’)-Ib-cr*, *aph(3’’)-Ib*, *aph(3’)-Ia*, *aph(6)-Id*	*oqx*A/B, *mdf*A	*arr-3*, *cat*A2/B3, *dfr*A14, *fos*A, *mph*A, *qnr*B4, *sul*1/2, *tet*A/D
KEB2	ECL (1)	25	NDM-1	**DHA-1** ACT-6		*oqx*A/B, *mdf*A	
KEC1	EC (1)	167	NDM-5	CTX-M-15, OXA-1, TEM-1B	***rmt*B**, *aac(6’)-Ib-cr*, *aad*A2	*mdf*A	*cat*B3, *dfr*A12, *mph*A, *sul*1, *tet*A
KEC2	EC (1)	167	NDM-5	CTX-M-15, EC, OXA-1	*aac(6’)-Ib-cr5*, *aad*A5	*acr*F, *mdt*M, *emr*D	*ble*, *dfr*A17, *mph*A, *sul*1, *tet*A
KEC3	EC (1)	648	NDM-5	CTX-M-15, EC, OXA-1, TEM-1	*aac(3)-Iia*, *aac(6’)-Ib-cr5*, *aac(3)-Iia*, *aac(6’)-Ib-cr5*, *aad*A5	*acr*F, *mdt*M, *emr*D	*ble*, *cat*A1, *dfr*A17, *mph*A, *sul*1, *tet*B
KKPI	KP (1)	147	OXA-181	CTX-M-15, SHV-67, TEM-1B	***rmt*F,** *aac(6’)-Ib3*, *aph(3’’)-Ib*, *aph(6)-Id*	*mdf*A, *oqx*A/B	*arr-2*, *dfr*A14, *fos*A, *mph*A, *sul*2
KKP2	KP (1)	219	NDM-1	**CMY**, CTX-M-15, SHV-1	***rmt*C** *aac(6’)-Ib3*, *aad*A2, *aph(3’)-Ia*, *aph(3’’)-Ib*, *aph(6)-Id*	*oqx*A10/B5, *kde*A, *emr*D	*ble*, *dfr*A12, *flo*R, *fos*A, *mph*A, *qnr*S1, *sul*1*/*2, *tet*A
KPA2-5	PA (4)	357	NDM-1, OXA-50	OXA-10, PAO,VEB-1	*aph(3’)-Iib*, *aad*A1, *ant(2’’)-Ia*, *aac(6’)-Il*, *aph(3’)-VI*		*cat*B7, *fos*A, *sul*1, *cml*A1, *dfr*B2, *arr-3*, *tet*A
KPA9-13	PA (5)	357	NDM-1	OXA-10, OXA-846, PDC-11, VEB-9	*aac(6’)-Il*, *aad*A1, *aac(6’)-Il*, *ant(2’’)-Ia*, *aph(3’)-Iib*, *aph(3’)-VI*	*mex*A/E/X	*arr-3*, *ble*, *cat*B7, *cml*A5, *dfr*B2, *fos*A, *sul*1, *tet*A
KPA14	PA (1)	357	NDM-1	OXA-396, PDC-3	*ant(4’)-Iib*, *aph(3’’)-Ib*, *aph(3’)-Iib*, *aph(6)-Id*	*mex*E	*cat*B7, *fos*A, *sul*1
KPA6	PA (1)	654	NDM-1	OXA-396, PAO	*aph(3’’)-Ib*, *aph(6)-Id*, *ant(4’)-Iib*, *aph(3’)-Iib*		*cat*B7, *fos*A, *sul*1
KPA1	PA (1)	316	VIM-1	OXA-10, OXA-395, PAO	*aph(3’’)-Ib*, *aph(6)-Id*, *aph(3’)-Iib*, *aac(6’)-Ib3*		*cat*B7, *fos*A, *sul*1, *tet*G, *flo*R
KPA7	PA (1)	1203	VIM-6	OXA-10, OXA-395, PAO	*aph(3’)-Iib*, *aad*A1, *ant(2’’)-Ia*, *aac(6’)-Ib3*		*cat*B7, *fos*A, *sul*1, *qnr*VC1, *dfr*A5, *dfr*B5, *ere*A
KPA8	PA (1)	1203	VIM-6	OXA-10, OXA-395, PAO	*aph(3’)-Iib*, *aad*A1, *ant(2’’)-Ia*, *aac(6’)-Ib3*		*cat*B7, *fos*A, *sul*1, *qnr*VC1, *dfr*A5/B5, *ere*A

PA, *P*. *aeruginosa*; KP, *K*. *pneumoniae*; EC, *E*. *coli*; ECL, *E*. *cloacae*; AB, *A*. *baumannii*. Antibiotic resistance gene families: **AmpC:** CMY, ADC-25, ACT-16, DHA-1, **ESBL:** CTX-M, SHV, TEM. Antibiotic resistance gene targets: **Bleomycin-***ble*; **Rifampicin**-*arr-2*, *arr-3*; **Chloramphenicols-***cat*A1, *cat*A2,*cat*B3, *cat*B7, *cml*A5, *cml*A1, *flo*R; **Fosfomycin**-*fos*A;**Tetracylines**- *tet*A, B, D, G, and 39; **Sulfonamides—***sul*1and 2; **Erythromycin—***ere*A; **Trimethoprim—***dfr*A1, A5, A12, A14, A17, A20, B2, and B5; **Quinolones—***qnr*VC1, S1, and B4; **Macrolides***—msr*E, *mph*A, and E; **Streptothricin—***sat*2.

*AmpC genes are highlighted **in bold**.

^#^The16srRNA methyltransferases are highlighted **in bold.**

The oxacillinases were the most abundant carbapenemases (23/79, 29.1%) detected mostly in *A*. *baumannii* isolates. Among the *A*. *baumannii* isolates, the predominant genes were *bla*_OXA-23, 66, and 69_, with only a few isolates bearing *bla*_OXA-58_ *and bla*_OXA-91_. The *bla*_OXA-181_ and *bla*_OXA-50_ genes were detected in *K*. *pneumonia* and *P*. *aeruginosa* isolates, respectively. Among the Metallo- β–lactamases, *bla*_NDM1_ (21/79, 26.6%) was detected in *A*. *baumannii*, *E*. *cloacae*, *P*. *aeruginosa*, *and K*. *pneumoniae* species. *bla*_VIM-1_ and *bla*_VIM-6_ genes were detected in three *P*. *aeruginosa isolates* and *bla*_NDM-5_ genes in three *E*. *coli* isolates. The greatest diversity of carbapenemases was found among *A*. *baumannii* (*bla*_NDM-1,_ *bla*_OXA-23, 58, 66, 69, 91_,) and *P*. *aeruginosa* (*bla*_VIM-1, 6,_ *bla*_NDM-1,_ *bla*_OXA-50_) (Table 3). Although most isolates had a single carbapenemase gene, five *A*. *baumannii* isolates (*bla*_OXA-23_/ *bla*_NDM-1_ (KAB13, 25, 26, 27) and *bla*_NDM1_/*bla*_OXA-58_ (KAB 14) and four *P*. *aeruginosa* isolates (KPA2-5) (*bla*_NDM-1,_ *bla*_OXA-50_) (Table 4) bore both a Class B and D carbapenemase gene. Genes for AmpC were detected among 17/27 *A*. *baumannii* (*bla*_ADC-25_), *E*. *cloacae* (*bla*_DHA-1_ and *bla*_ACT-16_), and *K*. *pneumoniae* (*bla*_CMY_) isolates (Table 4).

### Detection of other antimicrobial resistance genes among C-NS isolates

Given the phenotypic evidence of multidrug resistance in the C-NS isolates, it was unsurprising to find resistance genes against all significant antibiotic classes. Aminoglycoside resistance genes (*aph*, *aac*, *aad*, *ade*, *acr*, and *ant gene families*) were detected among all but one *E*. *cloacae* and three *A*. *baumannii* isolates. Apart from these genes specific to different aminoglycosides, the potent acquired 16srRNA methyltransferase genes (*arm*A, *rmt*A, *rmt*C, *and rmt*F), which confer pan-aminoglycoside resistance, were detected among *A*. *baumannii*, *E*. *cloacae*, *K*. *pneumoniae*, *and E*. *coli* (Table 4). These genes render this drug class virtually ineffective in the treatment of C-NS infections. Sulfonamide resistance genes (*sul*1/2) were detected in all isolates but one *E*. *cloacae* and two *A*. *baumannii isolates*. Various macrolide (*mph*E, *msr*E, *mph*A, *ere*A*)*, trimethoprim *(dfr*A *and* B*)*, chloramphenicol (*cat*A and B, *flo*R, *cm*1) and tetracycline genes (*tet*A, B, D, G, 39) were detected across all species (Table 4). Less frequently identified were resistance genes for quinolone, rifampin, and fosfomycin, as described in Table 4. Genes for efflux pumps that contribute to multidrug resistance (*amv*A, *oqx*A/B, *mdf*A, *mex*A, E, and X, *kde*A, *acr*F, *emr*D) were also detected, particularly among *E*. *cloacae*, *P*. *aeruginosa*, *E*. *coli*, and *K*. *pneumoniae* isolates.

### Sequence types and geographical distribution of the C-NS isolates

**Fig 1:**
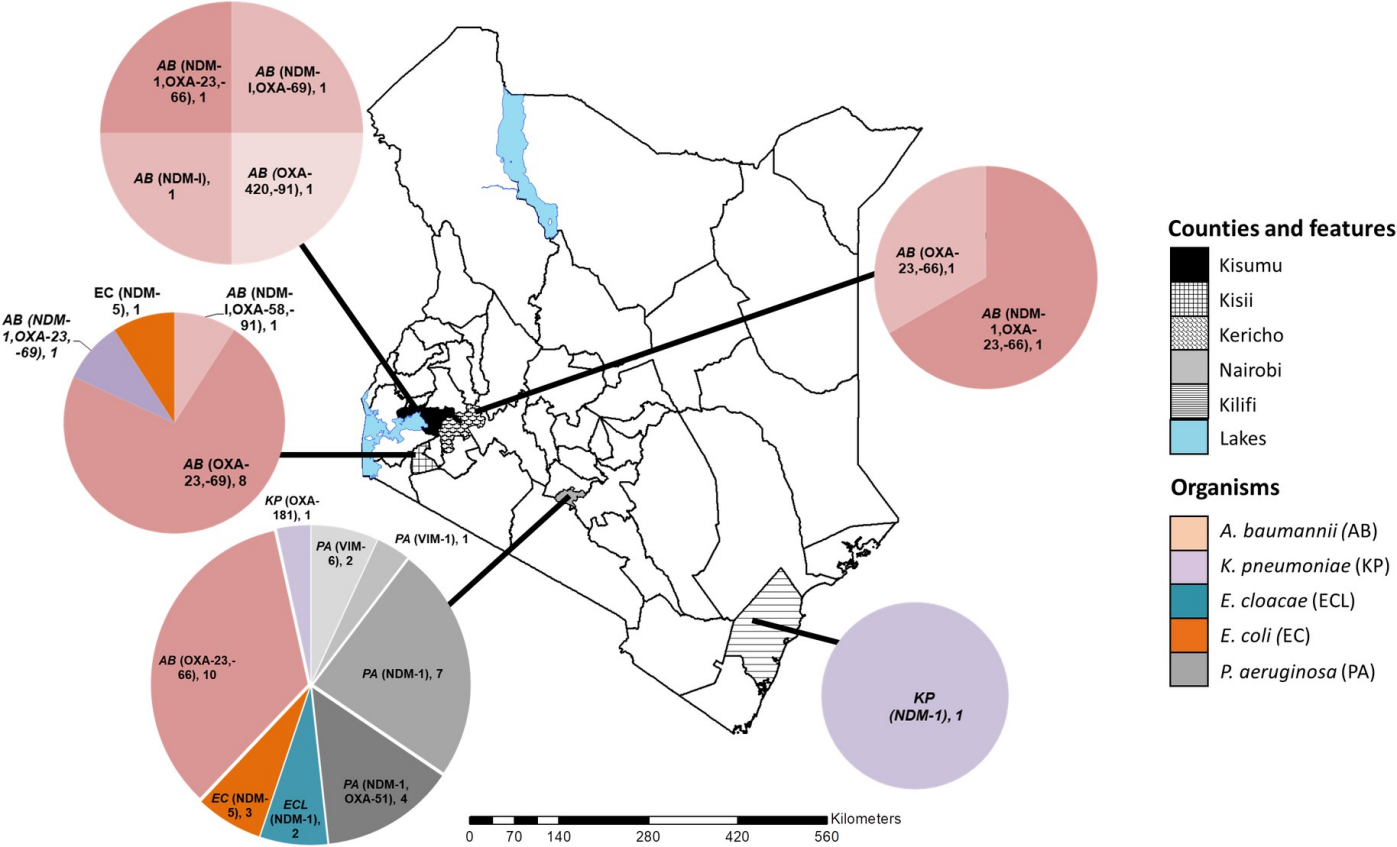
The geographical distribution of the carbapenem non-susceptible gram-negative bacteria and carbapenemase genes in five Kenyan counties. The map of Kenya with the study counties highlighted. The pie charts indicate the distribution of bacterial species, carbapenemase genes identified and the number of each isolate type detected. This custom map was generated in-house using the ArcGIS software Version 10.3.1 (Environmental Systems Research Institute, Red-lands, CA, USA).

Carbapenemases were identified in all the study counties. However, the geographical distribution of the CR genes, bacterial species, and strain types were variable (Fig 1). Among the C-NS bacterial species, *A*. *baumannii* was the most broadly distributed across the counties, while *E*. *cloacae* and *P*. *aeruginosa* were only detected in Nairobi. Kisii and Nairobi counties had the most types of carbapenemases. The bacterial strain types and their characteristics varied between counties as described for each species in the following section.

#### Acinetobacter baumannii

The C-NS *A*. *baumannii* isolates belonged to four STs: 1, 2, 164, and a novel type. The isolate with a novel ST possessed a new single variant allele of the *rpl*B gene (rplB 213) and was assigned to ST1475. In this study, all twelve ST1 *A*. *baumannii* isolates were detected in counties in western Kenya. They are predominantly associated with community-acquired infections (CAI) and were the least drug-resistant of the *A*. *baumannii* STs (Fig 1 and Table 1).

The ST1 isolates were mostly susceptible to fluoroquinolones, tetracyclines, tigecycline, and minocycline. A majority of ST1 isolates had the *bla*_OXA-23_ and *bla*_OXA-69_ carbapenemase and the *bla*_ADC-25_ genes. The exception was one isolate (KAB16), which had the *bla*_NDM-1_ carbapenemase, *bla*_CARB-16,_ and genes not found in the other ST1 isolates conferring resistance to macrolides, aminoglycosides, and fluoroquinolones. In contrast, the eleven ST2 isolates were multidrug-resistant, predominantly hospital-associated (8/11), and originated from the same hospital in Nairobi (10/11). These ST2 isolates expressed the *bla*_OXA-23_ and *bla*_OXA-66_ carbapenemase, the AmpC gene *bla*_ADC-25,_ and the 16s rRNA methyltransferase *armA* (Table 2), among other resistance genes.

The ST164 *A*. *baumannii* isolates (KAB 14, 15) differed in antibiotic resistance profiles and gene composition. The community-acquired KAB14 from Kisii had the *bla*_NDM1_ and *bla*_OXA-58_ carbapenemases and more resistance genes than KAB15 from Kisumu. This healthcare-associated KAB15 had fewer resistance genes and the *bla*_OXA-420_ carbapenemase gene. The ST374 isolate (KAB8) from an HAI in Kisumu was multidrug-resistant and unique because it lacked the oxacillinases. The ST1475 isolate (KAB13) associated with *bla*_NDM1_ and *bla*_OXA-23_ was isolated from an HA-SSTI infection in Kisii. The isolate was susceptible to only the last line drug tigecycline representing a localized high-risk MDR strain.

#### Enterobacter cloacae

The two *E*. *cloacae* isolates, both isolated from Nairobi, belonged to ST182 and ST25. They expressed the *bla*_NDM1_ carbapenemase, *bla*_DHA-1_, *bla*_ACT-6,_ *mdf*A, *oqx*A, *oqx*B genes, and were associated with HAIs. ST25 (KEB2) was the least resistant isolate, susceptible to levofloxacin and tigecycline. ST182 (KEB1**)** was resistant to all drugs tested except tigecycline. It had many drug resistance genes, including the potent 16S methyltransferase *rmt*C contributing to the highly drug-resistant phenotype.

#### Escherichia coli

All three *E*. *coli* isolates harbored *bla*_NDM-5_. However, the isolates from Nairobi (KEC1, 2) were ST167, while the Kisii isolate (KEC3) was ST648. All the *E*. *coli* isolates were only susceptible to tigecycline among the tested antibiotics, although ST648 had more antibiotic-resistant genes than the ST167 isolates implying a greater resistance level.

#### Klebsiella pneumoniae

ST219 and ST147 *K*. *pneumoniae* isolates were identified in Kilifi and Nairobi hospitals (Fig 1), respectively. The ST147 isolate from a CAI expressed a *bla*_OXA-181_ carbapenemase and was resistant to all drugs tested. On the other hand, *K*. *pneumoniae* ST219 had the *bla*_*NDM-1*_, *bla*_CMY,_ and the 16S methyltransferase *rmt*C genes. It was susceptible to levofloxacin, minocycline, and tigecycline.

#### Pseudomonas aeruginosa

Thirteen of the fourteen *P*. *aeruginosa* isolates were resistant to all antibiotics tested. Four ST types, 357, 654, 316, and 1203 –were identified among C-NS *P*. *aeruginosa* isolates from Nairobi. All ST357 isolates and the single ST654 isolate (KPA6) had the *bla*_NDM1_ carbapenemase. The single *P*. *aeruginosa* ST316 isolate, for which there was limited demographic information available, expressed *bla*_VIM-2_. Of the two isolates identified as ST1203 (KPA 7, 8), both had the *bla*_VIM-6_ gene and genes not identified in any of the other STs of *P*. *aeruginosa* in this study: *qnr*VC1, *dfr*A5, *dfr*B5, *ere*A genes.

## Discussion

This study aimed to identify the diversity and distribution of carbapenemase genes among GNB bacteria from an ongoing surveillance study in six hospitals across Kenya. The analysis detected eleven different carbapenemases among five clinically significant bacteria species. Five of the eleven CR genes detected were previously undescribed in Kenya: *bla*_OXA-420, 58,181_, *bla*_NDM-5,_ and *bla*_VIM-6_. Notably, *bla*_VIM-6_ has not been reported before in Africa since it was first observed in Singapore in 2004 [35] and subsequently spread across Asia [36]. Neither has *bla*_NDM-5_ been reported in East Africa. Other carbapenemase genes identified are naturally occurring or widespread in their species, such as the class D oxacillinases *bla*_OXA-23_ and *bla*_OXA-66 and 69_ in *A*. *baumannii* [37]. One ST374 isolate (KAB8) was unique because it lacked these intrinsic oxacillinases.

Previous studies on carbapenemases in Kenya have focused mostly on Enterobacteriaceae [18]. Our findings show that *A*. *baumannii* was the most abundant and widely distributed of the C-NS bacteria carrying approximately 72% of the CR genes detected. It was followed by *P*. *aeruginosa*, with approximately 19%. The two species had the most carbapenemase gene diversity. As *A*. *baumannii* and *P*. *aeruginosa* are significant causes of HAI, their carriage of CR genes could contribute to hard-to-treat hospital outbreaks across broad geographical settings in Kenya—a challenge the World Health Organization (WHO) recognizes [38]. A substantial number of infections were among inpatients with SSTI and catheter-associated UTIs. These indicators stood out as potential risk factors for the acquisition of C-NS infections. Surprisingly, a third of the infections caused by C-NS bacteria were CAI, consistent with the trend witnessed in other parts of the world [39] deviating from the previously strong association of C-NS infections with HAIs. To avoid missing community circulation of C-NS bacteria, CAI should be included in surveillance efforts.

This study also detected various AmpC genes (*bla*_ADC-25_, *bla*_CMY_, *bla*_DHA-1,_ and *bla*_ACT-16_), which have not previously been reported to coexist with carbapenemase genes in Kenya, and ESBL genes (*bla*_CTX-M_, _SHV, and TEM_). Hyper-production of these genes combined with mutations in porin genes or efflux pumps that reduce the influx or increase the efflux can contribute to the carbapenemase resistant phenotype [40]. It is necessary to include all these contributing factors in CR surveillance because, even in the absence of carbapenemases genes, they can cause carbapenem treatment failure.

Multiple antibiotic resistance genes (some of which were not phenotypically tested in this study) and efflux pumps (whose substrates can include antibiotics of multiple classes) coexisted in the C-NS bacteria. These data indicate the breadth of drug resistance and the therapeutic challenge posed by C-NS bacteria. Aminoglycosides are reported to be the third most prescribed drug class after cephalosporins and penicillins in a Kenyan referral hospital [41]. They are often used in combination with other antibiotic classes to treat severe infections. The high rate of aminoglycoside resistance, reflective of this prescription pattern, and the presence of the pan-aminoglycoside resistance genes among all isolates render this drug class ineffective against most C-NS infections. The 16srRNA methyltransferase genes have been previously associated with CR genes, particularly *bla*_NDM-1_ and *bla*_OXA_ [42, 43], similar to the findings in this study where 16srRNA methyltransferase was detected alongside *bla*_OXA-23/66_, *bla*_NDM-1_, *bla*_OXA-181,_ and *bla*_NDM-5_ genes.

The multidrug-resistant phenotypes in most C-NS isolates suggest an inevitable rise in untreatable GNB infections unless urgent measures are taken to curb these infections. This risk is evident in multiple MDR *A*. *baumannii* ST2 isolates identified in one hospital, indicating localized, clonal spread within the hospital. This pattern suggests a prolonged outbreak, as previously reported in a Nairobi Hospital [44]. Carbapenem, considered a last-resort antibiotic, is used to treat MDR infections and is the most commonly prescribed antibiotic in a study in a hospital in Nairobi [45]. As higher-level drugs are often unavailable or too expensive in Kenya, C-NS infections pose a serious challenge. This study shows that the most effective drugs against C-NS infections are tigecycline and minocycline. However, the innate resistance of *P*. *aeruginosa* and two *A*. *baumannii* isolates resistant to both drugs further limit the therapeutic options.

Few studies in Kenya have identified the strain types of C-NS bacteria. This study has set the baseline for tracking and detecting C-NS STs in Kenya by identifying the C-NS bacteria’s strain types. For example, the identification of *A*. *baumannii* STs (1, 2, 164, and a novel ST1475 type) was different from the OXA-23 producing strains identified in a study in a referral hospital in Kenya (ST110, 92, and 109) [44] indicating the broad strain diversity in the C-NS *A*. *baumannii* population in Kenya. *A*. *baumannii* ST1 and ST2 are global clonal strains associated with multidrug resistance [46]. ST164 is a rare strain type recently reported in Sudan, Brazil, and Turkey [47]. The ST164 *A*. *baumannii* bearing *bla*_OXA-58_ and *bla*_OXA-420_ carbapenemases are typically plasmid-mediated and geographically limited to Europe [48], Tunisia [49], and Nepal [50]. These ST164 isolates that carry potentially plasmid-mediated CR genes could be efficient disseminators of CR and MDR among the community and hospital settings in which they were found in Kenya. The *A*. *baumannii* ST1475 was previously un-typed, representing a novel strain first identified in Kenya. Carbapenem-resistant *P*. *aeruginosa* (CRPA) isolates carrying *bla*_VIM-2_ have been identified in an un-typed Kenya isolate [26] and ST244 and ST640 isolates in Tanzania and throughout Africa, often occurring in outbreaks [51–56]. Other CRPA ST316 isolates have been identified in China expressing the IMP-9 gene [57], but the isolate in this study represents a new case of a *bla*_VIM-2_ ST316 CRPA.

This study observed that different strain types had different drug resistance profiles making it easier to identify the most important strains for close monitoring. Based on the resistance profiles of the CR strains, the study identified high-risk MDR strains of *A*. *baumannii* (ST1475, ST2), *E*. *cloacae* (ST182), *K*. *pneumoniae* (ST147), *P*. *aeruginosa* (ST357, 654), and *E*. *coli (*ST167, ST648). *A*. *baumannii* ST1475 is a recognized international high-risk clone associated with multidrug resistance and hyper-virulence (44). *P*. *aeruginosa* ST654 isolates have been identified in Europe [58, 59], Singapore [60], Tunisia [61], and South America [62] associated with KPC, VIM, and IMP carbapenemases. Apart from this study, an extensively drug-resistant ST654 *bla*_NDM1_-producing *P*. *aeruginosa* isolate has only been reported in Canada [63]. NDM-5 was first detected in 2011 in an *E*. *coli* ST648 strain from India described as a highly virulent and MDR strain [64]. Since then, NDM-5 has been identified globally in different *E*. *coli* and *K*. *pneumoniae* STs indicating the rapid horizontal transfer of the gene across species. *E*. *coli* ST167 is a globally disseminated clone in human and animal populations associated with both multiple resistance and hyper-virulence genes. NDM-5 harboring strains have been described in Europe [65, 66] and China from humans [67]and poultry [68]. The potential zoonotic spread of these *bla*_NDM-5_ *E*. *coli* ST167 and ST648 strains in the Kenyan community poses a considerable risk to animal and human populations. The high-risk *bla*_NDM-1_ MDR *E*. *cloacae* ST182 isolate has been associated with outbreaks in several countries related to transmissible plasmid-borne *bla*_NDM-1_ [65, 69, 70]. These high-risk strains warrant close monitoring in Kenya as they could act as disseminators of carbapenemases.

An example of the potential or spread of carbapenemase genes is the increasing detection of *bla*_NDM-1_, previously identified in *K*. *pneumoniae*, *A*. *baumannii*, *and P*. *aeruginosa* from Nairobi and Kilifi [22–24, 71, 72]. This distribution is mirrored by this study’s findings of *bla*_NDM-1_ *K*. *pneumoniae and P*. *aeruginosa* in the two counties. However, what was different is that *bla*_NDM-1_ was also detected in *E*. *cloacae* isolates *(*Nairobi) and in *A*. *baumannii (*Nairobi, Kisii, and Kericho), an indication of a broader distribution of the CR gene. There have been few reports to date of *P*. *aeruginosa* expressing *bla*_NDM-1_ in sub-Saharan Africa and only one in Kenya [71]. It was surprising that five of the eight *P*. *aeruginosa* isolates in this study had *bla*_NDM-1_ as *P*. *aeruginosa* is most often associated with the Metallo-β lactamases VIM and IMP [73]. But NDM-1 expressing strains, typically associated with ST235, are increasing worldwide since they were first reported in 2011 [74] in Europe [75], India [76], Northern Africa [77] and North America [63] and may also become widespread in Kenya.

The study outcomes suggest that differences in CR’s type and scale among bacterial species exist between hospitals and counties. The greatest number and diversity of C-NS isolates were observed in the two referral hospitals in Kisii and Nairobi. Referral hospitals have factors that contribute to a greater risk of MDR infections, such as more hospital-acquired infections, extended hospitalization stays, invasive devices, and more frequent use of third-and fourth-line drugs [45]. These factors could explain why more carbapenemase genes were detected in Nairobi and Kisii hospitals than in the other lower-level hospitals with less than four carbapenem genes.

The study had some limitations. Only forty eight isolates representing all the available carbapenemase isolates detected over the study period were studied. Additional C-NS GNB isolates will be explored in the future within this ongoing surveillance program to improve the understanding of the geospatial distribution, emergence, spread, and evolution of CR in Kenya. The susceptibility testing was performed on a limited panel of antibiotics on the VITEK2 platform, and the AST results were not confirmed using an independent method. However, the detection of carbapenemase and other antibiotic resistance genes by WGS confirmed the observed resistance phenotypes. The primary concern with CR is the possibility of transmissible versus chromosomal carbapenemases. In this study, detailed analysis to detect the genomic location of the CR genes (chromosomal or plasmid) was not performed as it exceeded its scope, whose main goal was to describe the presence and distribution of carbapenemase genes. These analyses will be the focus of future studies to evaluate the dissemination risks within and between bacterial species.

## Conclusions

In conclusion, while CR and the presence of carbapenemase genes have been recognized in Kenya, this study expands our understanding in several ways outlined below.

First, the study has described the diversity of CR genes among clinically significant gram-negative bacteria, including for the first time in *E*. *coli* and *Enterobacter* spp. Second, the research has shown the wide geographical spread of CR across Kenya, including three counties (Kisumu, Kisii, and Kericho) not previously studied. Third, the study provides the first report of carbapenemases OXA-420, 58,181, and VIM-6 and the simultaneous carriage of both carbapenemase genes and AmpC genes in Kenyan isolates. Fourth, CR is identified as a more significant challenge in larger referral hospitals where risk factors such as numbers of inpatient and critical care populations and greater use of third- and fourth-line antibiotics exist. Fifth, the study has made clear that across all hospitals, C-NS *A*. *baumannii* warrants attention as its significance in the CR landscape was not previously appreciated. Sixth, the notion that CR is only a nosocomial challenge is dispelled by the C-NS GNB detected in both community and healthcare-associated isolates. Finally, the study has identified STs of globally disseminated high-risk and multidrug-resistant isolates that should be specifically targeted for close monitoring. These isolates are reservoirs and possible transmitters of multiple-drug resistance.

These data highlight the importance of CR surveillance to adequately measure the scale of the problem and identify high-risk strains and emerging resistance genes to track the spread of resistance. Based on this data, measures such as improving infection control and implementing antibiotic stewardship should be implemented with the urgency required to reduce the spread of resistance, limit the morbidity and mortality associated with carbapenem resistance, and to preserve this critical drug class in Kenya.
